# Next-generation sequencing for HLA typing of class I loci

**DOI:** 10.1186/1471-2164-12-42

**Published:** 2011-01-18

**Authors:** Rachel L Erlich, Xiaoming Jia, Scott Anderson, Eric Banks, Xiaojiang Gao, Mary Carrington, Namrata Gupta, Mark A DePristo, Matthew R Henn, Niall J Lennon, Paul IW de Bakker

**Affiliations:** 1 Broad Institute of MIT and Harvard, Cambridge, Massachusetts, USA; 2 Harvard-MIT Division of Health Sciences and Technology, Boston, Massachusetts, USA; 3 Cancer and Inflammation Program, Laboratory of Experimental Immunology, SAIC-Frederick Inc., National Cancer Institute, Frederick, Maryland 21702, USA; 4 Ragon Institute of Massachusetts General Hospital, Massachusetts Institute of Technology and Harvard University, Boston, Massachusetts 02114, USA; 5 Division of Genetics, Department of Medicine, Brigham and Women's Hospital, Harvard Medical School, Boston, Massachusetts, USA; 6 Department of Medical Genetics, University Medical Center Utrecht, Utrecht, The Netherlands; 7 Julius Center for Health Sciences and Primary Care, University Medical Center Utrecht, Utrecht, The Netherlands

## Abstract

**Background:**

Comprehensive sequence characterization across the MHC is important for successful organ transplantation and genetic association studies. To this end, we have developed an automated sample preparation, molecular barcoding and multiplexing protocol for the amplification and sequence-determination of class I HLA loci. We have coupled this process to a novel HLA calling algorithm to determine the most likely pair of alleles at each locus.

**Results:**

We have benchmarked our protocol with 270 HapMap individuals from four worldwide populations with 96.4% accuracy at 4-digit resolution. A variation of this initial protocol, more suitable for large sample sizes, in which molecular barcodes are added during PCR rather than library construction, was tested on 95 HapMap individuals with 98.6% accuracy at 4-digit resolution.

**Conclusions:**

Next-generation sequencing on the 454 FLX Titanium platform is a reliable, efficient, and scalable technology for HLA typing.

## Background

The major histocompatibility complex (MHC) region on the short arm of chromosome 6 is one of the most complex regions in the human genome with extreme levels of polymorphism and linkage disequilibrium [1-3]. With a span of about 4 Mb, the MHC comprises many hundreds of genes [4]. Of these, the human leukocyte antigen (HLA) genes are the most prominently studied. The HLA genes encode cell-surface proteins responsible for antigen peptide presentation in a cell-mediated immune response. Inherited DNA sequence variation within these genes is strongly associated with autoimmune and infectious diseases as well as severe adverse drug reactions [5-8]. Clinically, HLA sequence information is also widely used for matching donor and recipient in transplantation on the basis that more similar alleles will reduce the risk of rejection [9].

To date, 2048 unique 4-digit HLA alleles have been described in the IMGT/HLA database at class I, and 751 at class II [10]. Over many years, best practices in HLA typing have traditionally been disseminated by the participants of the International Histocompatibility Workshop. Established methods include sequence specific oligonucleotide (SSO) hybridization and, more recently, capillary sequencing (Sanger method). SSO hybridization uses oligonucleotide probes to detect the presence (or absence) of polymorphisms specific to each probe[11]. The Sanger method uses chain-termination fluorescence for DNA base-pair detection and sequencing.

Although these methods have proven effective for HLA typing, they remain labor-intensive, time-consuming and expensive. Further, one specific disadvantage of Sanger sequencing is that it does not generate two separate, haploid sequences, making it in some cases challenging to resolve the individual HLA haplotype sequences in a diploid pair of chromosome 6. The advent of next-generation sequencing technologies motivated us to develop an efficient protocol for genotyping of the classical HLA genes of class I.

**Figure 1:**
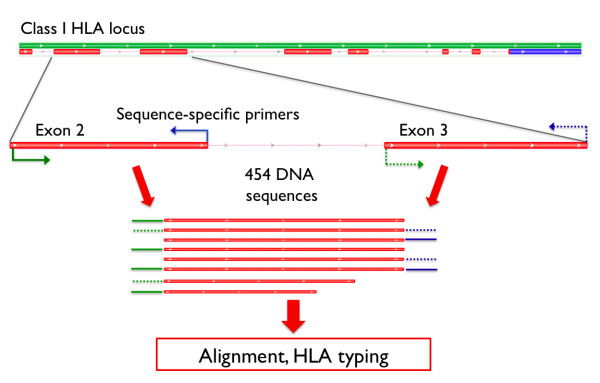
**HLA Class I amplification strategy**. PCR amplification of the polymorphic exons 2 and 3 in the class I HLA loci was performed (using primers within introns surrounding each exon) prior to 454 sequencing.

Our strategy for HLA typing utilizes the 454 GS FLX Titanium sequencing platform (Roche) and allows the use of sequence tags, or barcodes, to label each DNA sample at either the amplicon-preparation stage (using PCR primers tailed with a molecular barcode) or during library construction (using barcoded adaptors) (Figure 1, Additional File 1).

The first method, termed "library construction-based barcoding," involves the addition of a molecular barcode to the standard 454 "A" adapter, which is ligated to double-stranded exon-specific amplicons during library construction. The exon-specific amplicons are pooled by sample (six products per pool) after PCR, and up to 96 samples are pooled after barcoded adapter ligation (Additional File 1).

The second method, termed "PCR-based barcoding" involves the addition of a barcode to the forward and reverse exon-specific PCR primers. The same unique barcode is added to all 6 exon-specific primer pairs (exons 2 and 3 of HLA-A, -B and -C) for a given sample. 95 different barcoded primer sets were designed in total, leaving an empty 96^th ^well as a positional key (Additional File 1). Post-PCR all amplicons from all 95 samples are pooled and the pool proceeds through standard library construction with the addition of a non-barcoded adapter.

Both methods facilitate sample multiplexing prior to emulsion PCR and sequencing, which dramatically reduces the overall cost per individual for an all-in cost of less than $40 per sample for typing of Class I genes HLA-A, -B and -C. Using these methods a single technician can process up to 96 samples at once, creating a sequence-ready library in under three days. Because we are using the FLX Titanium chemistry, sequence reads extend across entire exons of the HLA genes (~350 base pairs).

**Figure 2:**
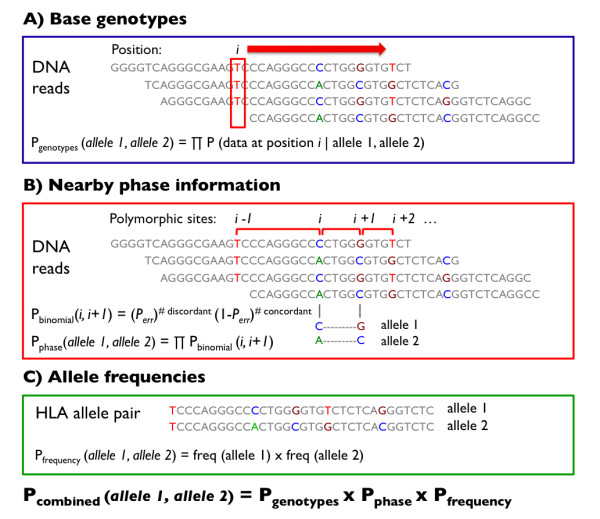
**Schematic of the HLA Caller Algorithm**. The HLA calling algorithm determines the most likely pair of HLA types at each locus by systematically evaluating all possible pairs of 4-digit HLA types. A) The genotyping algorithm within the GATK calculates the probability of observing particular genotypes in the data given a pair of HLA alleles. Probabilities were combined multiplicatively across base positions to obtain the cumulative probability based on genotypes. B) A binomial distribution function was used to calculate of probability of observing particular haplotypes in the data given a pair of HLA alleles. Probabilities were combined multiplicatively across pairs of polymorphic positions to obtain the cumulative probability based phase information. C) Prior probabilities for specific allele pairs were calculated as the product of allele frequencies in a specific population. Probabilities based on genotypes, phase information, and allele frequencies were combined multiplicatively to obtain the posterior probability for each HLA allele pair.

In parallel, we have developed an HLA calling algorithm to process sequence reads, from the now-standard SAM/BAM format, and to infer classical types for a given DNA sample (Figure 2)[12]. Recognizing that the improvements in next-generation sequencing technologies are rapid, we designed the HLA caller to be an integral part of the Genome Analysis Toolkit (GATK), a data processing tool for recalibration, quality control, and variant calling of next-generation sequence data [13].

The HLA calling algorithm determines the most likely pair of HLA types at each locus by systematically evaluating all possible pairs of 4-digit HLA types. We use three key components to calculate the posterior probability for each HLA allele pair. First, we compare genotypes for each allele pair to the genotypes determined by the Genome Analysis Toolkit (GATK) based on sequence data. Second, we check the allelic phase of each HLA allele pair for consistency with the sequence data. Specifically, we calculate the binomial probability that the phase orientation for a specific HLA allele pair is consistent with the sequence data at a pair of adjacent polymorphic sites, and aggregate these probabilities across all pairs of polymorphic sites. Third, we use information about the expected allele frequency to determine the prior probability of observing each pair of HLA alleles in the population (if the ancestry is known). We then multiply the probabilities calculated from base genotypes, allelic phase information, and allele frequencies, rescale (to ensure all posteriors sum to 1), and output the posterior probability for each HLA allele pair. The pair with the highest posterior probability corresponds to the best-guess genotype for that DNA sample.

In this study, we benchmark our protocol on DNA samples used in the International HapMap Project with known HLA types: 270 samples for library construction-based barcoding and 95 samples for the barcoded PCR method. We limited the sample number in our validation test of the barcoded PCR method to 95 due to the similarities to the already tested library-construction based method, and the number of barcoded primers arrayed per plate (i.e. 95 barcodes plus 1 empty well). We demonstrate that we can generate reliable HLA calls, and in some cases improve upon the existing calls, but also highlight instances of problematic alleles where calls are less robust in our current protocol. Overall, our protocol offers comparable data quality but outperforms traditional Sanger sequencing in terms of cost-effectiveness and throughput.

## Results

Using the library construction-based barcoding method, we sequenced exons 2 and 3 of *HLA-A*, *HLA-B *and *HLA-C *for each of 270 HapMap individuals (http://hapmap.ncbi.nlm.nih.gov/) (Table 1). This generated a total of 7.4 × 10^6 ^base-pairs of sequence data in 203,108 sequencing reads. The average read length across all libraries was 364 ± 124 bp (Additional file 2). 97.1% of reads were parsed using uniquely identifying barcodes, of which 94.2% were correctly aligned to the reference genome (Additional File 2). The average depth of coverage across all samples was 103 ± 65 reads at each exon (Table 2 indicates coverage by plate (set of 96) and by individual exon). Depth of coverage plots per individual and per locus for the library construction method are shown in Additional Files 3, 4 and 5.

**Table 1:** Distribution of HapMap samples by population and plate sequenced

	Plate 1	Plate 2	Plate 3	Total
CEU	0	90	0	90

YRI	27	6	57	90

JPT	24	0	21	45

CHB	45	0	0	45

**Total**	**96**	**96**	**78**	**270**

**Table 2:** Depth of coverage per individual across loci and plate pools

Gene	Exon	Plate 1 Coverage	Plate 2 Coverage	Plate 3 Coverage	Average coverage per locus
HLA-A	2	159 ± 87	65 ± 37	91 ± 60	106 ± 76

HLA-A	3	144 ± 61	71 ± 36	101 ± 52	106 ± 59

HLA-B	2	177 ± 74	89 ± 50	105 ± 56	125 ± 73

HLA-B	3	159 ± 72	93 ± 45	111 ± 54	121 ± 65

HLA-C	2	85 ± 44	62 ± 44	81 ± 66	76 ± 52

HLA-C	3	102 ± 50	70 ± 36	84 ± 50	85 ± 47

Average coverage per plate		137 ± 74	75 ± 43	95 ± 58	103 ± 65

**Figure 3:**
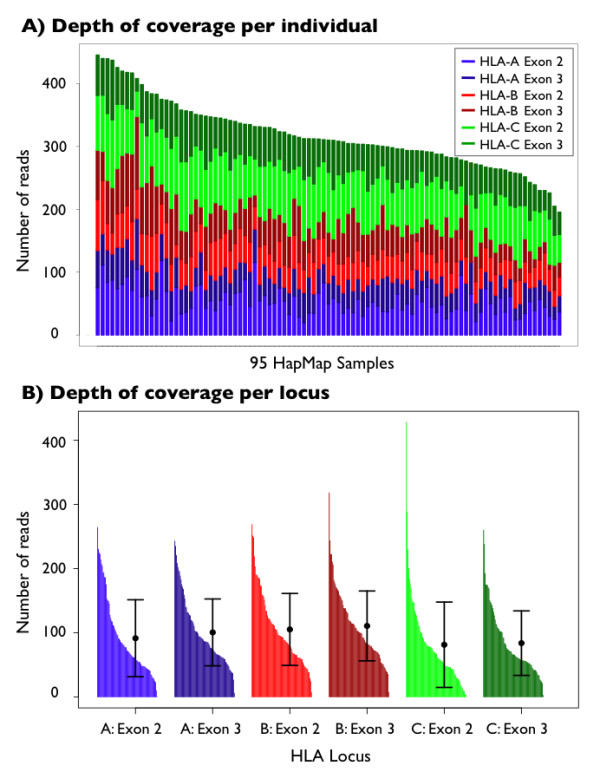
**Depth of coverage for 95 HapMap individuals**. Sequenced using the barcoded-PCR method: (a) coverage by individual, and (b) coverage across each of 6 exons.

**Figure 4:**
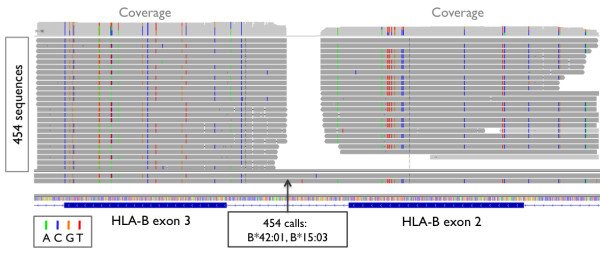
**Integrative Genomics Viewer (IGV) sequence data**. IGV snapshot of sequence data at HLA-B for one HapMap sample (NA18507), and the corresponding calls made from the data. Each horizontal bar represents one read, and colored bars indicate where DNA base pairs differ from the reference.

Using the PCR-Based Barcoding method, we sequenced class I loci for 95 HapMap samples and produced a total of 45,398 reads. 91.3% of reads were parsed using uniquely identifying barcodes, of which 97.5% were correctly aligned to the reference genome (Additional File 6). The average depth of coverage per individual was 53 ± 21 reads at each exon. Coverage plots for the barcoded-PCR method are shown in Figure 3 and an example of aligned DNA reads at HLA-B is shown in Figure 4.

The gold standard data set of 270 HapMap individuals generated using SSO hybridization contained 1,540 HLA types at 4-digit resolution[11]. Using data from the library construction-based barcoding method, we found that 1446 (93.9%) of our HLA calls were concordant with the gold standard types (Table 3). Of the 94 discordant cases, 38 included systematic errors in the gold standard dataset (see below), 49 resulted from inaccurate typing due to low or unbalanced chromosomal coverage, and 7 were inaccurately typed due to sample contamination, sequencing errors, or read misalignment.

**Table 3:** HLA calling accuracy and concordance with gold standard in 270 individuals

Population	Concordant alleles	Original type missing	Original type incorrect	Incorrect call due to low coverage	Incorrect due to other reasons	Total	Accuracy
CEU	457	66	6	10	1	540 (= 90 × 6)	97.7% (463/474)

YRI	485	8	16	29	2	540 (= 90 × 6)	94.2% (501/532)

JPT	246	6	9	5	4	270 (= 45 × 6)	96.6% (255/264)

CHB	258	0	7	5	0	270 (= 45 × 6)	98.1% (265/270)

**Total**	**1446**	**80**	**38**	**49**	**7**	**1620**	**96.4%**

**Figure 5:**
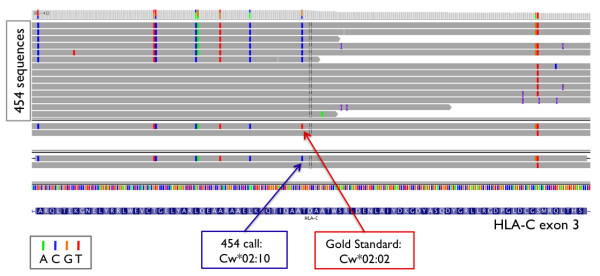
**IGV snapshot: Example 1**. An example of HLA-C*02:10 mislabeled as C*02:02 in the gold standard (NA15822).

**Figure 6:**
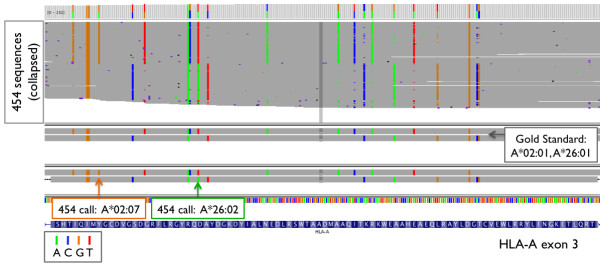
**IGV snapshot: Example 2**. IGV snapshot: an example of HLA-A*02:07 mistyped as A*02:01, and A*26:02 mistyped as A*26:01 in the same individual (NA18987) in the gold standard. The orange and green arrows indicate the polymorphisms that differ between the respective types.

Manual inspection of the aligned reads from the library construction-based barcoding method revealed that inconsistent use of HLA allele nomenclature between the gold standard and our HLA caller (based on the official HLA dictionary), as well as typing errors in the gold standard generated by SSO hybridization, accounted for 38 of 94 (40%) of the discordant HLA calls. In the gold standard, the C*02:10 genotype was consistently misnamed as C*02:02 in 11 cases (Figure 5) since this genotype was previously named Cw*020204 (which was removed in May 2006 after it was found to be identical to C*02:10 [14,15]). In other instances, genotype A*02:07 was consistently mistyped as A*02:01 in 10 cases in the gold standard data, because an A > T transversion at position 30,019,048 of HLA-A exon 3 (that defines the difference between the two 4-digit genotypes) was missed (Figure 6). Similarly, genotypes C*08:03, C*07:01, A*66:03, A*26:02, and A*36:03 were each mistyped in 2 individuals due to single point mutations not detected in the HLA types of the gold standard data. Lastly, C*06:06, C*05:01, C*03:04, C*01:03, B*39:24, B*39:06, and A*11:02 were each mistyped once due to differences at one or more bases (Table 4). In total, the detected error rate of the gold standard was 2.3% (38 of 270 × 6 genotypes) at the 4-digit resolution. All corrections were reviewed in detail with the laboratory that performed the original SSO experiments (X.G. and M.C.), and were independently confirmed using Sanger sequencing. The corrected class I HLA types are listed in Additional File 7.

**Table 4:** HLA alleles originally mistyped in the gold standard dataset

Population	Occurrences	454 type	Original type	Difference(s)
YRI	11	C*02:10	C*02:02	31346962 T>C

CHB/JPT	10	A*02:07	A*02:01	30019048 A>T

JPT	2	C*08:03	C*08:01	31346853 C>T

CEU	2	C*07:01	C*07:02	31347428 C>G

YRI	2	A*66:03	A*66:02	30018721 G>C

YRI	2	A*36:03	A*36:01	30019043 A>G

JPT	2	A*26:02	A*26:01	30018098 G>A

CEU	1	C*06:06	C*06:02	31346910 A>G

CEU	1	C*05:01	C*16:01	31347486 A>T
				31347396 A>T
				31347522 A>T

CEU	1	C*03:04	C*03:03	31347355 C>T

JPT	1	C*01:03	C*01:02	31347029 T>A
				31347036 A>T

YRI	1	B*39:24	B*39:03	31432177 G>A

CEU	1	B*39:06	B*39:05	31432180 G>C
				31432185 C>G
				31432186 C>A
				31432187 C>G
				31432188 C>G
				31432495 C>A

JPT	1	A*11:02	A*11:01	30018566 A>G

After taking into account errors in the gold standard dataset, overall typing accuracy in the library construction-based barcoding method increased to 96.4% at 4-digit resolution and 98.8% at 2-digit resolution (Table 5). 4-digit accuracy was higher in *HLA-B *(99.4%) compared to *HLA-A *(95.9%) and *HLA-C *(94.4%) (Table 5). Typing accuracy was higher in CEU (97.7%), JPT (96.6%), and CHB (98.1%) individuals compared to YRI (94.2%)(Table 3).

**Table 5:** HLA typing accuracy across class I loci in 270 HapMap individuals using the library construction method

Gene	4-digit accuracy	2-digit accuracy
HLA-A	**95.9%** 497/518	**99.2%** 522/526

HLA-B	**99.4%** 499/502	**99.4%** 513/516

HLA-C	**94.4%** 488/517	**97.8%** 506/517

All loci	**96.4%** 1,484/1,540	**98.8%** 1,541/1,559

**Figure 7:**
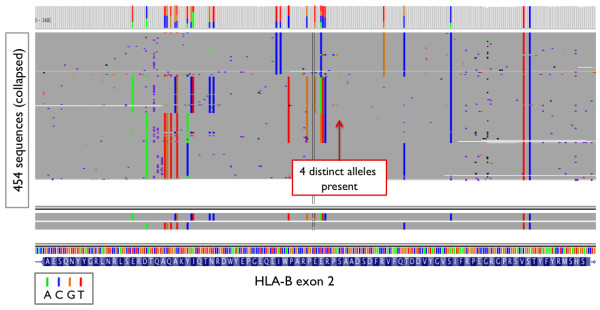
**IGV snapshot: Example 3**. IGV snapshot of a sample (NA18970) miscalled due to presence of 4 distinct alleles/chromosomes in the data. Other loci also showed similar patterns.

Most of the incorrect calls in the library construction-based barcoding method were due to low coverage of specific alleles (Table 6). In these cases, unbalanced chromosomal coverage caused the HLA caller to mistake true heterozygous sites for homozygous bases (an example is shown in Additional File 8). Overall, mistyping at the 4-digit resolution due to low or unbalanced chromosomal coverage occurred in 3% (49 of 270 × 6) of all cases. Sample contamination contributed to mistyping in one individual in the library construction method, as evidenced by the presence of four distinct chromosomes at a single locus (Figure 7). This individual was later correctly typed using the PCR based barcode method.

**Table 6:** HLA alleles mistyped in 454 sequencing due to low or unbalanced coverage

Population	HLA allele	# Miscalls	# Correct calls
YRI/CEU	C*04:01	24	35

YRI	A*30:01	8	25

CHB	A*31:01	4	16

JPT/CHB	A*33:03	3	33

YRI	A*30:02	2	7

JPT	C*07:02	1	78

YRI	C*02:10	1	10

CHB	B*46:01	1	15

CEU	B*35:01	1	29

CEU	A*29:02	1	8

CEU	A*11:01	1	32

In the PCR-based barcoding method, 95 individuals were sequenced and typed, with a typing accuracy of 98.6% at 4-digit resolution and 99.6% at 2-digit resolution after adjusting for errors in the gold standard (Additional File 9). The improved typing accuracy resulted from reducing uneven coverage of specific HLA alleles. C*04:01, the genotype with the poorest coverage in the original method, was typed correctly in all 28 individuals with that genotype using this method. Likewise, A*30:01 was typed correctly in all 9 individuals with the genotype, highlighting the reduction of errors due to low coverage using this method.

## Discussion

We have developed two sample preparation methods and a systematic algorithm for class I HLA typing using next-generation sequencing. These methods leverage the longer read lengths and clonal amplification inherent to the 454 sequencing technology with the Titanium chemistry. Longer reads (>300 bp) capture critical phase information of nearby DNA variants, and enable accurate sequence alignment to the polymorphic HLA region. This information enables differentiation between highly similar alleles and is an important advantage over capillary-based methods.

The ability to capture DNA sequences with high depth of coverage and with phase information enabled us to confidently detect and correct errors in the gold standard database, originally typed using SSO hybridization. The high depth of coverage of 454 sequences minimizes genotyping errors, and the long read lengths and use of locus-specific primer sequences together help prevent alignment errors. Compared to SSO hybridization, 454 holds two main advantages: first, 454 generates many DNA reads for each chromosome while SSO generates only one intensity-based read, allowing 454 to correct for sequencing errors while SSO cannot. Second, reads from 454 sequencing contain phase information that can be used to resolve ambiguous allele combinations while SSO reads cannot. These advantages allowed us to confidently correct errors in the SSO based gold standard, and corrections were verified the laboratory that performed the original SSO experiments.

Related methods for performing HLA typing using 454 sequencing were recently developed by Gabriel et al., Lank et al. and Bentley et al. [16-18]. All of these methods employ a PCR primer modified with both barcode and adaptor sequences [16-18], resulting in significantly more expensive primers (requiring HPLC cleanup) for DNA library amplification than those in our method. In addition, we multiplex 95-96 barcoded samples into a single lane of an 8-region PicoTiter Plate (PTP), allowing for a maximum of 760 samples per run compared to 24 - 48 samples per run in the method by Bentley et al [16]. The current estimate for minimum coverage required for accurate HLA typing by our HLA caller is 20 reads, whereas our samples received on average >100 reads per exon. This leaves open the possibility of sequencing additional amplicons per sample, or multiplexing more samples per sequencing run in future if necessary. The longer read lengths provided by the FLX Titanium technology also permit more flexibility in primer selection than the FLX technology used in the Bentley et al study. See Additional File 10 for a comprehensive comparison of these methods to ours.

While sequencing HLA exons 2 and 3 is not sufficient for distinguishing between all known HLA genotypes, it provides an extraordinary amount of identifying information, especially when combined with allele frequency information. Using the HLA database, Lank et al. (2010) predict that exons 2, 3, and 4 theoretically differentiate 85% of all known alleles, however this figure likely underestimates the true typing capacity since they did not considering the effect of allele frequency on the incidence of uniquely identifiable alleles. We were able to achieve 96.4% to 98.6% empirical 4-digit typing accuracy by sequencing only exons 2 and 3, in addition to using frequency information to differentiate between ambiguous HLA genotypes. Without frequency information, it is difficult to identify the true HLA types, but it is possible to generate a short list (average 3 to 6) of candidate HLA allele pairs from a total of 34,584 possibilities at HLA-A, 143,648 at HLA-B, and 48,672 at HLA-C. The true allele pair may be differentiated from the other candidates by having the highest multiplicative frequency in the vast majority of cases. In the rare instance that the HLA genotype remains ambiguous after using frequency information, a list of top candidate alleles may be provided. Additional exons may be sequenced if high-resolution HLA types are desired, for to type individuals in rare populations or for to discover new HLA alleles.

One concern is that the incorporation of population frequency information may bias the HLA typing for a few specific HLA alleles. The definitive solution for preventing report bias is to sequence the entire HLA gene to remove all ambiguities in the genotype background. However this approach is not yet cost effective for most technologies and may hinder multiplexing throughput and efficiency. The interim solution is to report all alleles that match the particular genotype background (nucleotide sequence) and phase, with a pair of alleles that are designated to be the most likely given the expected frequency in the population in question. Another concern may be the accuracy of allele frequencies. All frequency information was collected from http://www.allelefrequencies.net, a central resource that has aggregated HLA type frequencies from hundreds of sources including blood banks, research facilities, and other studies [19]. To our knowledge, this is the most reliable source of HLA (and KIR) allele frequencies available. The problem of allele skew is common to all HLA typing methods that do not sequence the entire gene, however report bias may be prevented by reporting all matching alleles in addition to the most likely alleles with frequency information.

Our sequencing methods may be modified to accommodate additional amplicons at the HLA loci (i.e. class I exon 4, or exons in HLA class II). Because the average depth of coverage in the current method (> 100 reads per site) exceeds the estimated coverage required for accurate typing (10-20 reads), the addition of a small number of additional amplicons (i.e. exon 4 of HLA-A, -B, and/or -C) may work in the current multiplexing protocol. Efforts are also underway to develop class II HLA typing capacity. However, the addition of a significant number of amplicons (1 to 2 for each of HLA-DRB1, -DQA, -DQB, -DPA, -DPB) may require decreasing the multiplexing rate by about ½ to maintain a similar depth of coverage. However such changes are well compensated by the high efficiency of sample multiplexing and cost savings from pre-barcoded primers. The main challenge in class II is to design primers that target specific class II HLA genes instead of others in the same homologous family (HLA-DRB1, -DRB2, - DRB3, etc).

We have described two sample preparation methods that differ in the pooling and barcoding strategies employed. Both methods represent facile approaches to amplification and HLA Class I genotyping. If large sample numbers (thousands) are to be processed the PCR-based barcoding method may be preferential as it facilitates pooling of all amplicons directly after PCR, meaning that only a single library construction is required downstream. These changes significantly cut the cost and increase the throughput of HLA typing. Using this process a single technician can amplify, pool and adapt up to 96 samples in two days. Class I HLA typing of samples prepared in this way costs less than $40 per sample, as calculated using our all-in, end-to-end internal accounting model.

## Conclusions

Next-generation 454 sequencing is a reliable, efficient, and scalable technology for HLA typing. Important advantages of 454 over traditional sequencing technologies include its high depth of coverage and long read lengths to improve HLA typing accuracy, as well as its high multiplexing rate to maximize throughput while minimizing cost. We have developed novel laboratory methods to sequence the class I HLA loci using 454 technology, and a robust HLA caller algorithm to perform HLA typing using sequence genotype, allelic phase, and population-based frequency information. We have validated these laboratory and computational methods by typing HapMap individuals with high accuracy, and in some cases, provided corrections to the SSO-based gold standard database after systematic review and validation

## Methods

### HapMap samples

A set of 270 samples were selected from the original HapMap collection, and consisted of 90 Yoruban, 90 European, 45 Chinese and 45 Japanese individuals (Table 1). These samples were previously sequenced via SSO hybridization [2].

### Primer design

Six HLA Class I exonic regions were amplified by PCR: exons 2 and 3 in each of HLA-A, -B, and -C. Primers were designed to fall within the intronic regions surrounding the exon of interest (Figure 1, Additional File 11). Each primer pair was designed to uniquely target a specific HLA locus without promiscuity. Degenerate bases were included in primers to accommodate the known sequence diversity, as described in the international ImMunoGeneTics IMGT/HLA database (http://www.ebi.ac.uk/imgt/hla/) [10,20]. Primer sequences and locations are described in Additional File 11. Several resources were used for primer design: MUSCLE (http://www.ebi.ac.uk/Tools/muscle/) was used for multiple alignment, Jalview (http://www.jalview.org/) was used for alignment visualization, Primer3 (v 0.4.0, http://frodo.wi.mit.edu/primer3/) was used for primer sequence design, and In-Silico PCR (http://genome.ucsc.edu/cgi-bin/hgPcr?command=start) was used to ensure that each primer pair would amplify only a single region in the human genome [10,20-24].

### PCR amplification and Pooling

#### Library construction-based barcoding method

Six PCR amplifications were performed for each sample; one for each of the six exons of interest. Reaction setup was performed on the Bravo Liquid Handling Platform (Agilent Technologies, Santa Clara, CA). Briefly, 20 ul reaction volumes consisted of 1× Platinum Taq buffer (Invitrogen, Carlsbad, CA), 10 mM dNTPs, 50 mM MgSO4, 10 mg/ml BSA, 0.5% DMSO, 1 uM of each primer, 0.2 ul Platinum Taq (Invitrogen, Carlsbad, CA), with 100 ng genomic DNA. Thermal cycling conditions were: 3 mins at 95°C, 15 secs at 95°C × 5 cycles, 15 secs at 62°C, 60 secs at 72°C, 15 secs at 95°C × 26 cycles, 15 secs at 58°C, 60 secs at 72°C, 15 secs at 95°C × 4 cycles, 60 secs at 55°C, 2 mins at 72°C, and 7 mins at 72°C. PCR product sizes were confirmed on 2% eGels (Invitrogen, Carlsbad, CA) and, after 1:1 dilution of the PCR product in water, concentrations were determined using the Quant-it PicoGreen dsDNA Kit (Invitrogen, Carlsbad, CA). PCR products from the six amplicons for a given patient were normalized by concentration before being pooled together prior to library construction. Pooled, normalized PCR reactions were purified using 1.5× the PCR reaction volume of Agencourt AMPure XP beads (Beckman Coulter, Brea, CA).

#### Barcoded-PCR method

Six amplifications per sample were set up as described above with the only difference being that each primer pair for a given patient sample was tailed with a specific barcode tag sequence. Amplified products were normalized by concentration and all amplicons for every patient were pooled into a single receptacle. Pooled products were purified and concentrated using Qiagen's Minelute kit (Qiagen, Hilden, Germany) prior to library construction.

### Library Construction and 454 sequencing

Our library construction protocol was slightly modified from the GS FLX Titanium General Library Preparation Manual (October 2008) [25] to include process automation using the Bravo Liquid Handling Platform [26] (Additional File 1). Briefly, amplicons were made blunt-ended and phosphorylated using T4 polymerase (NEB, Ipswich, MA) and Polynucleotide Kinase (NEB, Ipswich, MA). 454 amplification and sequencing adaptors were then ligated to the resulting blunt-ends. To facilitate sample pooling for the library construction-based method, we designed a set of 120 modified adaptors, with each adaptor having a unique sequence tag immediately following the adaptor key sequence ([26] for more details). After adaptor ligation, ligase was heat inactivated at 65°C for 10 minutes, and samples were pooled by volume into a single tube. Each pool was concentrated and cleaned using a MinElute Reaction Cleanup Kit (Qiagen, Germany) and Ampure XP beads, (Beckman Coulter Genomics, Beverley, MA), prior to standard bead immobilization and strand-melting (GS FLX Titanium General Library Preparation Manual, October 2008). Adapted, single-stranded library pools were then quantified by qPCR, prior to emulsion PCR amplification and sequencing on a 454 Genome Sequencer FLX instrument (Additional File 1). For the 270 HapMap samples prepared using library construction-based barcoding, a total of three library pools were created, with 96 samples in each of the first two pools, and 78 in the third. All sequence data associated with this project has been submitted to NCBI's Short Reads Archive (SRA database, under accession number SRP003652.

### 454 read processing and alignment

DNA sequence reads for each pool were obtained in binary SFF format, and converted to FASTA/FASTQ format using the software SSFINFO (Roche). Reads for each individual were parsed using uniquely identifying 6-9 base-pair molecular barcodes. For each individual, reads corresponding to specific HLA loci were identified using the 18-21 base-pair primer sequences. Parsed reads were then aligned locally to the reference sequence at *HLA-A*, *HLA-B*, or *HLA-C *using SSAHA2 (http://www.sanger.ac.uk/resources/software/ssaha2/) and output in SAM/BAM format [alignment parameters included: word hash size = 8, step size = 3, tolerated difference from reference = 10]. Misalignments were detected algorithmically by comparing aligned reads to the dictionary of known HLA alleles at each locus (IMGT/HLA database). Reads identified with having < 75% SNP homology with the closest matching allele were removed. After misalignment removal, the average depth of coverage was calculated for each individual at each of the six exons amplified.

### HLA sequence dictionary and allele frequencies

DNA sequences for all HLA alleles were obtained from the IMGT/HLA database (http://www.ebi.ac.uk/imgt/hla/) version 2.26. Four-digit HLA genotype frequencies were obtained from http://www.allelefrequencies.net for "Caucasoid," "Black," and "Asian" populations. A weighted average of allele frequencies was calculated for each population using the numerous available sources available from this central repository for each population. The latest HLA nomenclature [27,28] was adopted for all typing purposes.

### Classical HLA genotype assignment

The HLA calling algorithm was developed using Java as part of the open-source Genome Analysis Tool Kit (GATK) [13]. The GATK can be found at http://www.broadinstitute.org/gsa/wiki/index.php/The_Genome_Analysis_Toolkit.

We use the HLA calling algorithm (Figure 2) to examine next generation sequencing data to determine the most likely HLA genotypes at each locus. Using this tool, we systematically evaluate all possible pairs of 4-digit HLA alleles and calculate the posterior probability for each allele pair based on three key considerations: 1) the genotypes at each base position observed across reads, 2) the phase information of neighbouring variants observed on individual reads, and 3) the frequency of different alleles at each locus. After calculating probabilities based on each of these key considerations, we multiply the scores to determine the overall posterior probability that an HLA allele pair is the true genotype.

We first use the GATK to determine the probability of observing each base genotype at every position in the HLA loci given the 454 sequence reads. The GATK automatically incorporates base and alignment quality scores when calculating these probabilities. For a pair of HLA alleles, we then iterate through each base position and multiply the probabilities of observing the base genotypes unique to these HLA alleles given the 454 sequence reads as previously calculated by the GATK (Figure 2, panel A). This is repeated for each HLA allele pair at every locus to determine *P_genotype_*.

We then use a phasing algorithm to examine each pair of adjacent polymorphic sites and, using a binomial distribution, calculate the probability that the phase orientation for a specific HLA allele pair is consistent with the sequence data at those sites (Figure 2, panel B). This probability is calculated by counting the number of sequence reads that match the allelic phase of the HLA allele pair in question compared to the total number of reads, and a binomial probability is determined using an estimated sequencing error rate (*P_err_*) of 1%. This probability is then aggregated multiplicatively across all adjacent polymorphic sites for each HLA allele pair, and is termed *P_phase_*.

We then calculate the probability of observing each pair of HLA alleles in the population by multiplying the two allele frequencies of interest (Figure 2, panel C). This probability was termed *P_frequency_*. We used allele frequencies corresponding to the White, Black, and Asian populations obtained from http://www.allelefrequencies.net.

Finally, we combine the three scores by multiplying *P_genotypes_, P_phase_*, and *P_frequency _*to determine the overall posterior probability, *P_combined_*, that a particular allele pair is the true type. We normalized these probabilities to ensure that the probabilities for each locus sum to 1. We report all allele pairs that match the particular genotype background and phase (i.e. that have the highest *P_genotypes _*× *P_phase_*), and designate a pair of alleles that are as the most likely given the addition of frequency information (i.e. that has the highest *P_combined_*). These alleles are assigned as the final HLA calls.

We determined two- and four-digit typing accuracy by comparing HLA calls to the gold standard types. All non-concordant calls were manually inspected using the Integrative Genomics Viewer (IGV, http://www.broadinstitute.org/igv/) to identify the possible reasons for discordance. Incorrect calls in the gold standard data were reported to the original typing laboratory, and were validated using Sanger sequencing.

The generated sequence data and HLA calls are available from the Resources section at: http://debakker.med.harvard.edu/

## Authors' contributions

RE designed the HLA sequencing protocol and all 454 sequencing experiments. XJ developed the HLA caller software and performed all post-experimental data processing, HLA typing, and data analysis. SA helped design and automate the high-throughput sequencing method. MH and NL provided intellectual and logistical support for the sequencing protocol. XG and MC helped review discrepancies between 454 calls and gold standard types. EB, MD, and PdB provided intellectual and logistical support for the HLA calling algorithm.

## Supplementary Material

Additional file 1 **Sample preparation processes for HLA typing**. Process map for (a) Library construction-based barcoding and (b) PCR-based barcoding. Click here for file

Additional file 2 **Data sequence characteristics generated from 270 HapMap individuals in three sample pools**. Click here for file

Additional file 3 **Depth of Coverage Plate 1**. Depth of coverage for 96 of 270 HapMap individuals sequenced on plate 1: (a) coverage by individual, and (b) coverage across each of 6 exons. Click here for file

Additional file 4 **Depth of Coverage Plate 2**. Depth of coverage for 96 of 270 HapMap individuals sequenced on plate 2: (a) coverage by individual, and (b) coverage across each of 6 exons. Click here for file

Additional file 5 **Depth of Coverage Plate 3**. Depth of coverage for 96 of 270 HapMap individuals sequenced on plate 3: (a) coverage by individual, and (b) coverage across each of 6 exons. Click here for file

Additional file 6 **Sequence metrics generated in pilot study for the barcoded-PCR method in 95 HapMap individuals pooled on a single plate**. Click here for file

Additional file 7 **Class I HLA types for 270 HapMap samples**. Click here for file

Additional file 8 **Example of mistyping**. IGV snapshot: an example of mistyping at HLA-A due to low 454 coverage of A*3001 (NA18507). Click here for file

Additional file 9 **Typing accuracy in 95 samples sequenced using the barcoded-PCR method**. Click here for file

Additional file 10 **454 HLA Typing Methods and their Attributes**. Click here for file

Additional file 11 **HLA class I primer sequences and locations**. Click here for file
