# Supplementary material for: Next-generation sequencing for HLA typing of class I loci
Source: BMC Genomics. 2011 Jan 18;12:42. doi: 10.1186/1471-2164-12-42 (PMC3033818; doi:10.1186/1471-2164-12-42)
Supplement: Additional file 1 — Sample preparation processes for HLA typing. Process map for (a) Library construction-based barcoding and (b) PCR-based barcoding. [file 1471-2164-12-42-S1.PPT]

## Slide 1
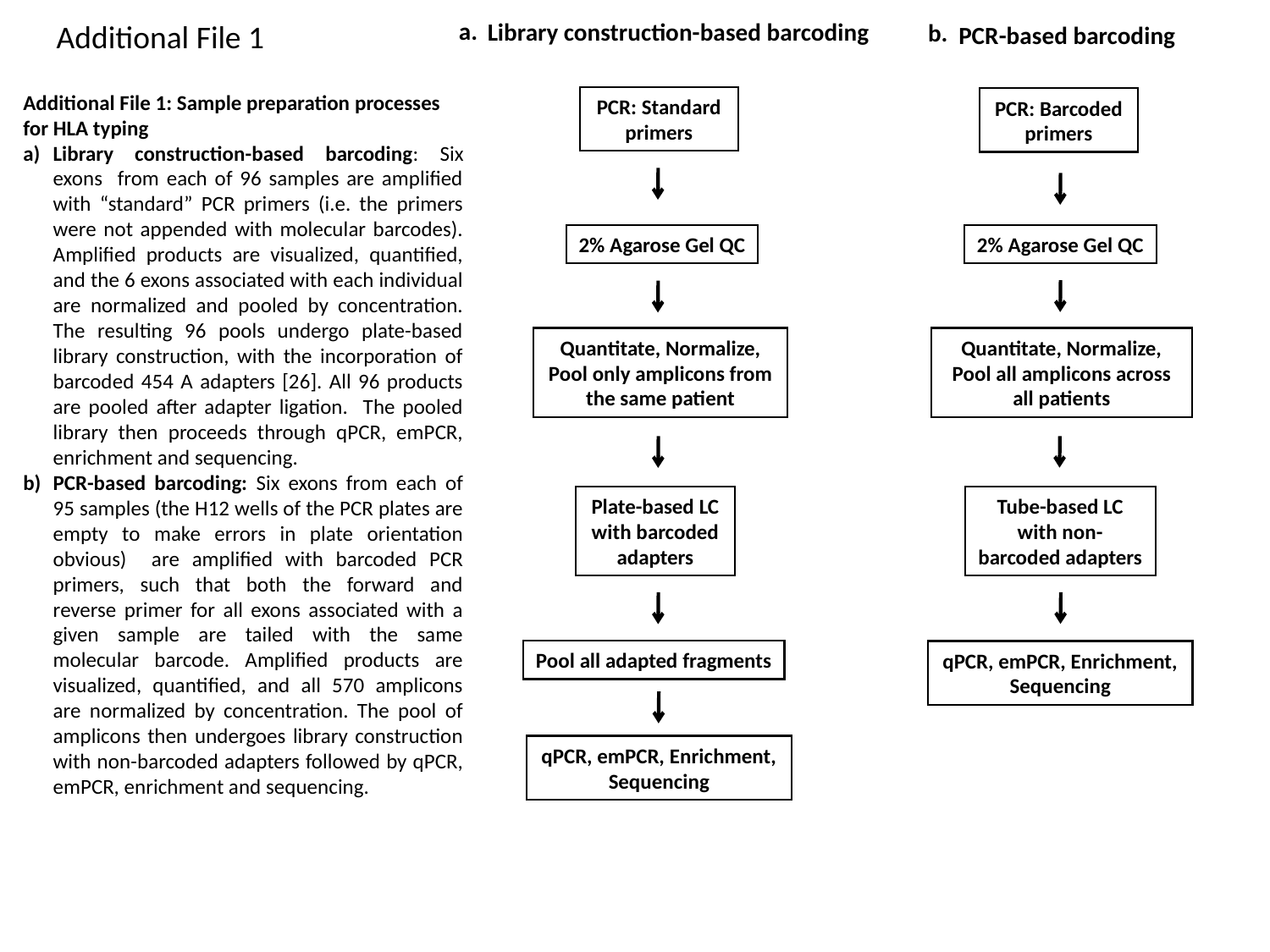

a.
Library construction-based barcoding
Additional File 1
b.
PCR-based barcoding
Additional File 1: Sample preparation processes for HLA typing
Library construction-based barcoding: Six exons from each of 96 samples are amplified with “standard” PCR primers (i.e. the primers were not appended with molecular barcodes). Amplified products are visualized, quantified, and the 6 exons associated with each individual are normalized and pooled by concentration. The resulting 96 pools undergo plate-based library construction, with the incorporation of barcoded 454 A adapters [26]. All 96 products are pooled after adapter ligation. The pooled library then proceeds through qPCR, emPCR, enrichment and sequencing.
PCR-based barcoding: Six exons from each of 95 samples (the H12 wells of the PCR plates are empty to make errors in plate orientation obvious) are amplified with barcoded PCR primers, such that both the forward and reverse primer for all exons associated with a given sample are tailed with the same molecular barcode. Amplified products are visualized, quantified, and all 570 amplicons are normalized by concentration. The pool of amplicons then undergoes library construction with non-barcoded adapters followed by qPCR, emPCR, enrichment and sequencing.
PCR: Standard primers
PCR: Barcoded primers
2% Agarose Gel QC
2% Agarose Gel QC
Quantitate, Normalize, Pool only amplicons from the same patient
Quantitate, Normalize, Pool all amplicons across all patients
Plate-based LC
with barcoded adapters
Tube-based LC with non-barcoded adapters
Pool all adapted fragments
qPCR, emPCR, Enrichment, Sequencing
qPCR, emPCR, Enrichment, Sequencing
