# Supplementary figures and images for: Next-generation sequencing for HLA typing of class I loci
Source: BMC Genomics. 2011 Jan 18;12:42. doi: 10.1186/1471-2164-12-42 (PMC3033818; doi:10.1186/1471-2164-12-42)

## Additional File 3

### A. Depth of coverage per individual

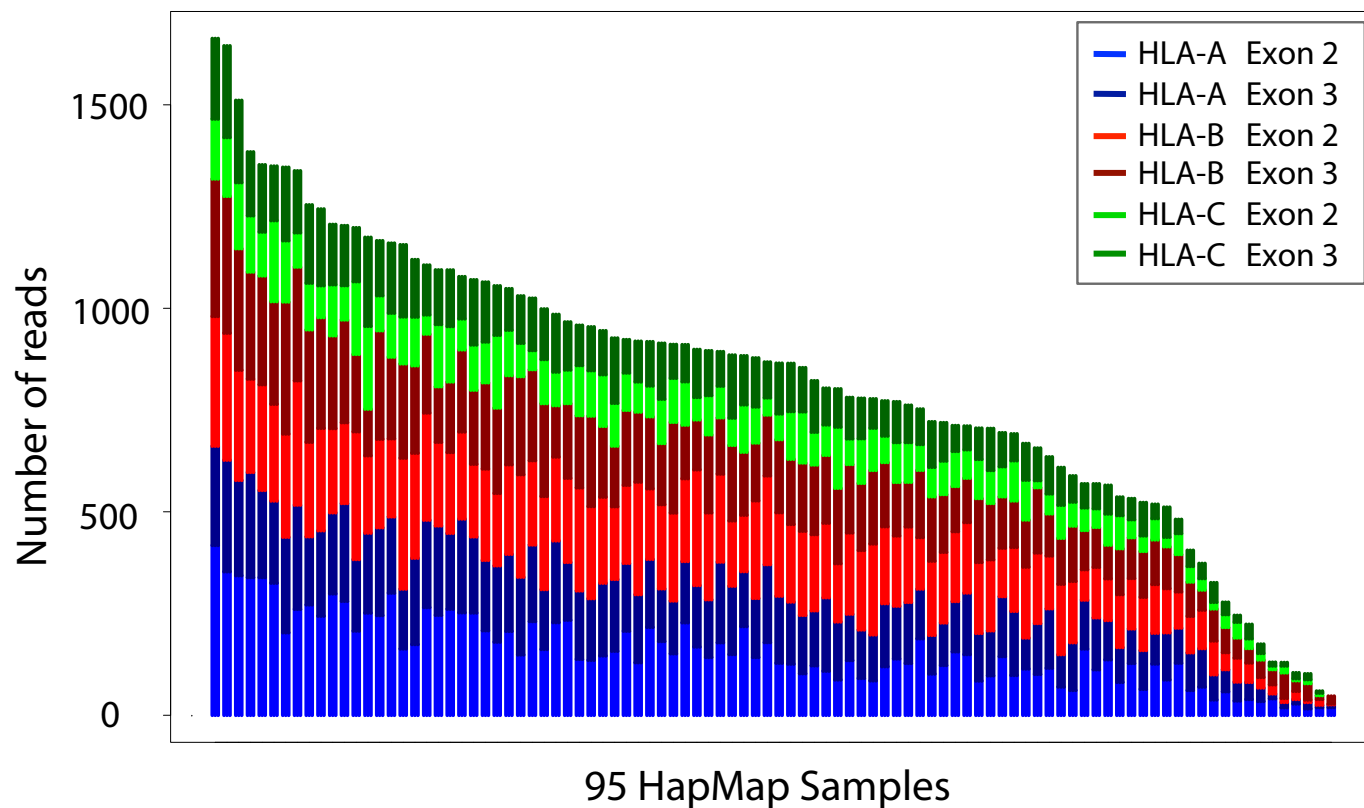

### B. Depth of coverage per locus

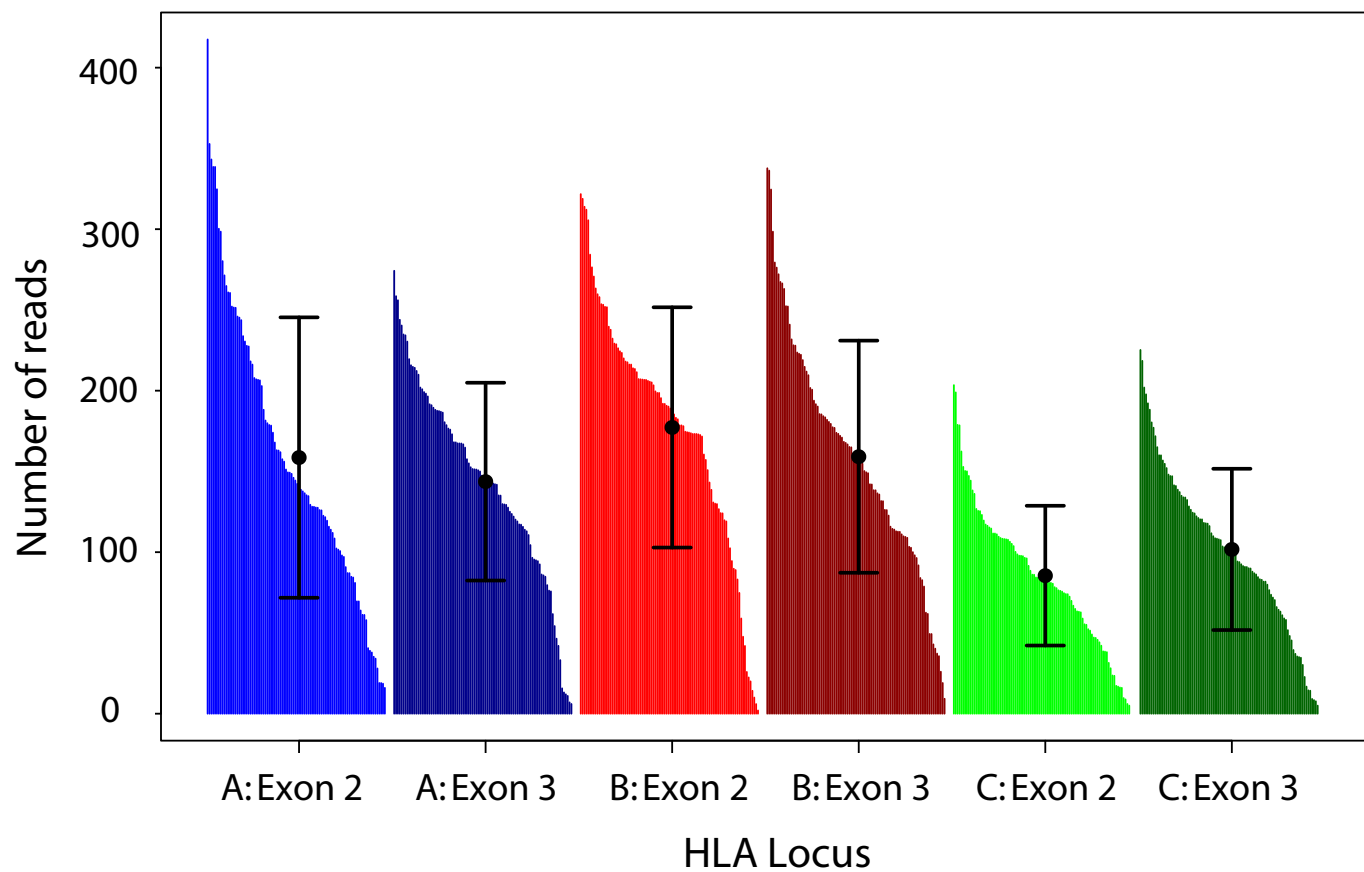

Supplement: Additional file 3 — Depth of Coverage Plate 1. Depth of coverage for 96 of 270 HapMap individuals sequenced on plate 1: (a) coverage by individual, and (b) coverage across each of 6 exons. [file 1471-2164-12-42-S3.PDF]

Additional File 4

A. Depth of coverage per individual

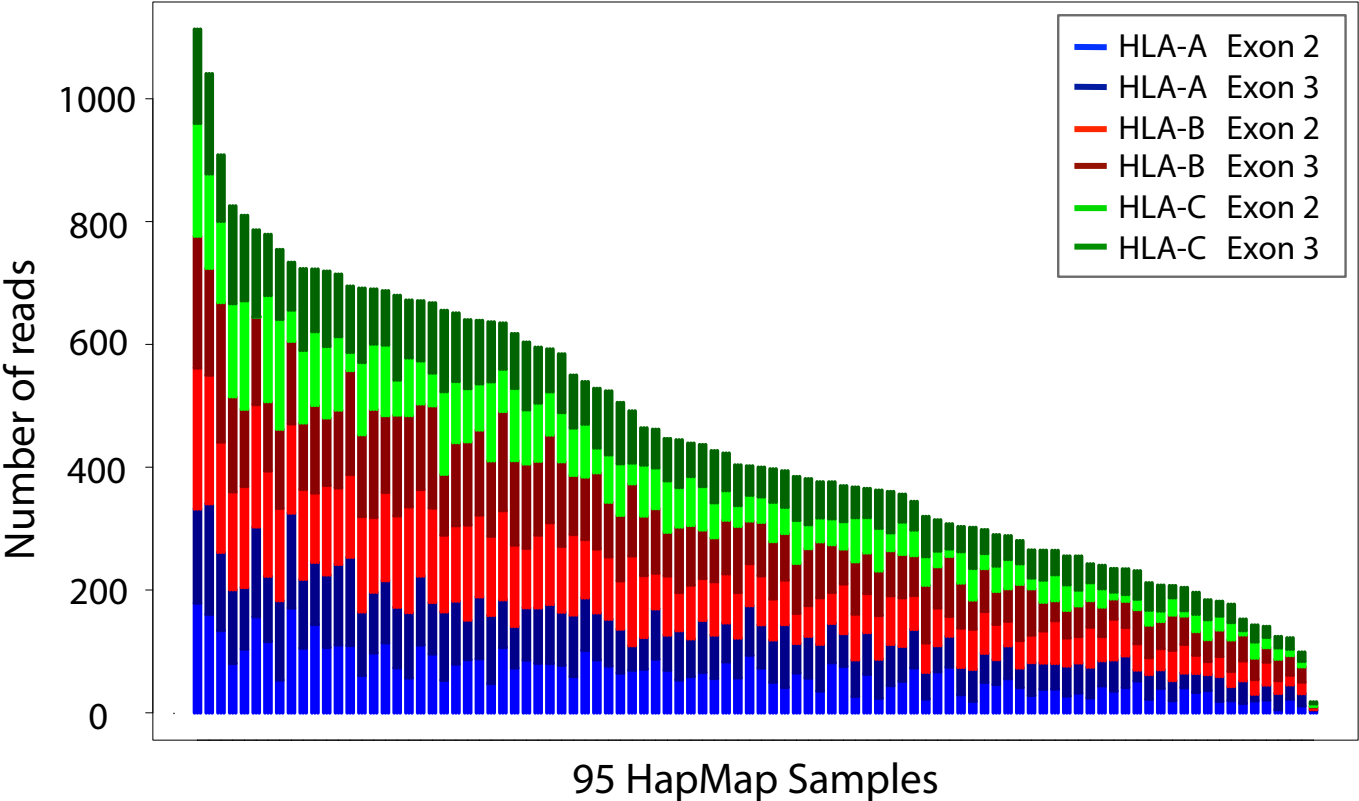

B. Depth of coverage per locus

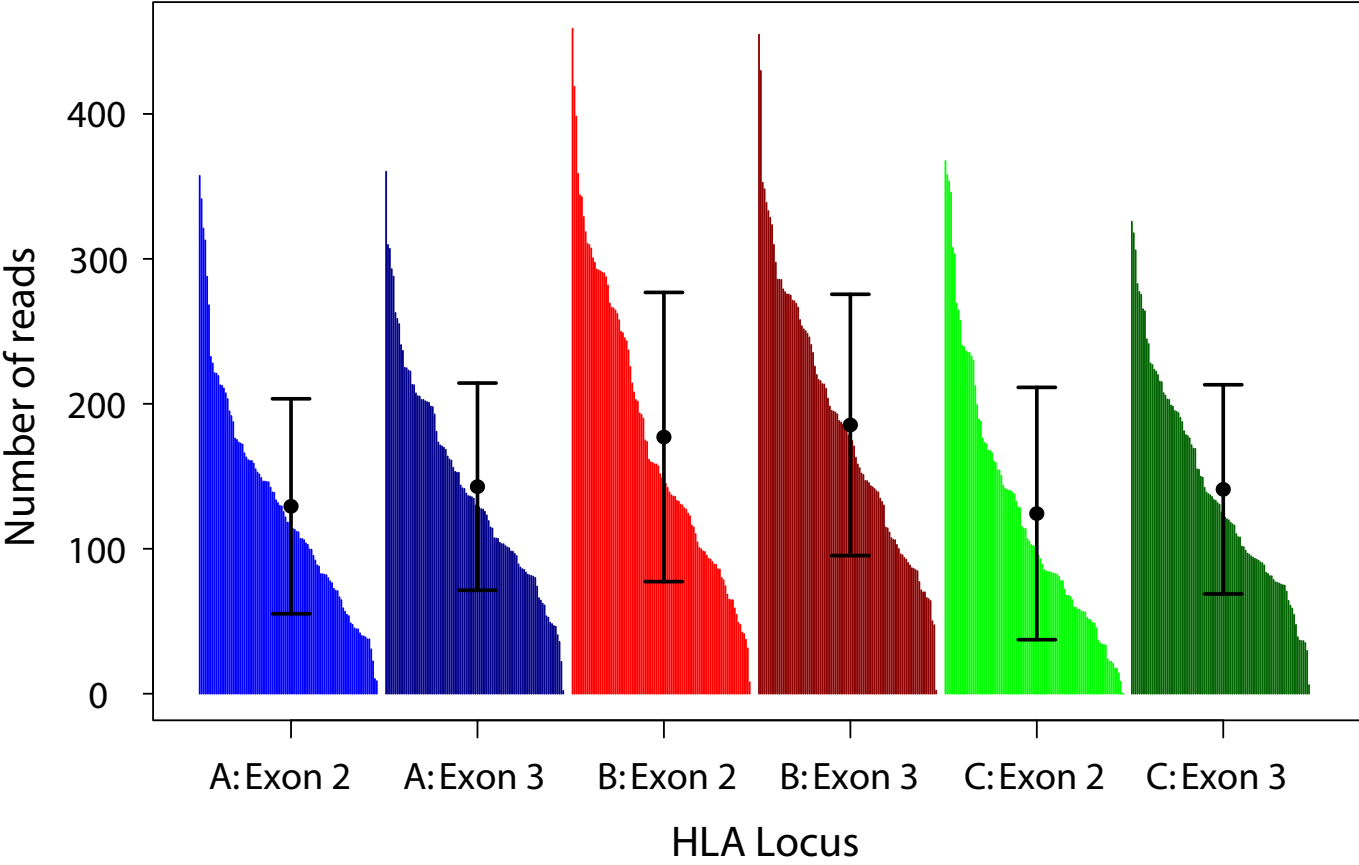

Supplement: Additional file 4 — Depth of Coverage Plate 2. Depth of coverage for 96 of 270 HapMap individuals sequenced on plate 2: (a) coverage by individual, and (b) coverage across each of 6 exons. [file 1471-2164-12-42-S4.PDF]

Additional File 5

A. Depth of coverage per individual

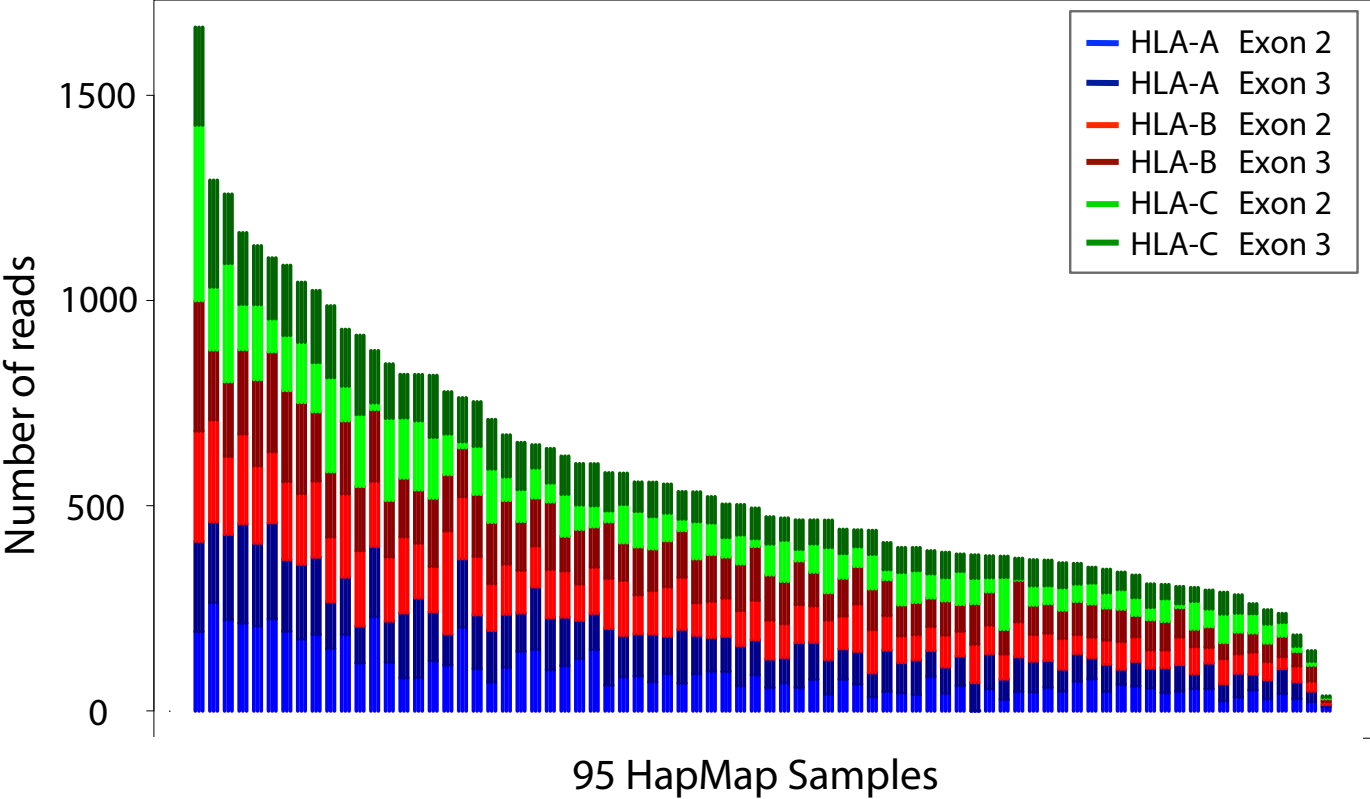

B. Depth of coverage per locus

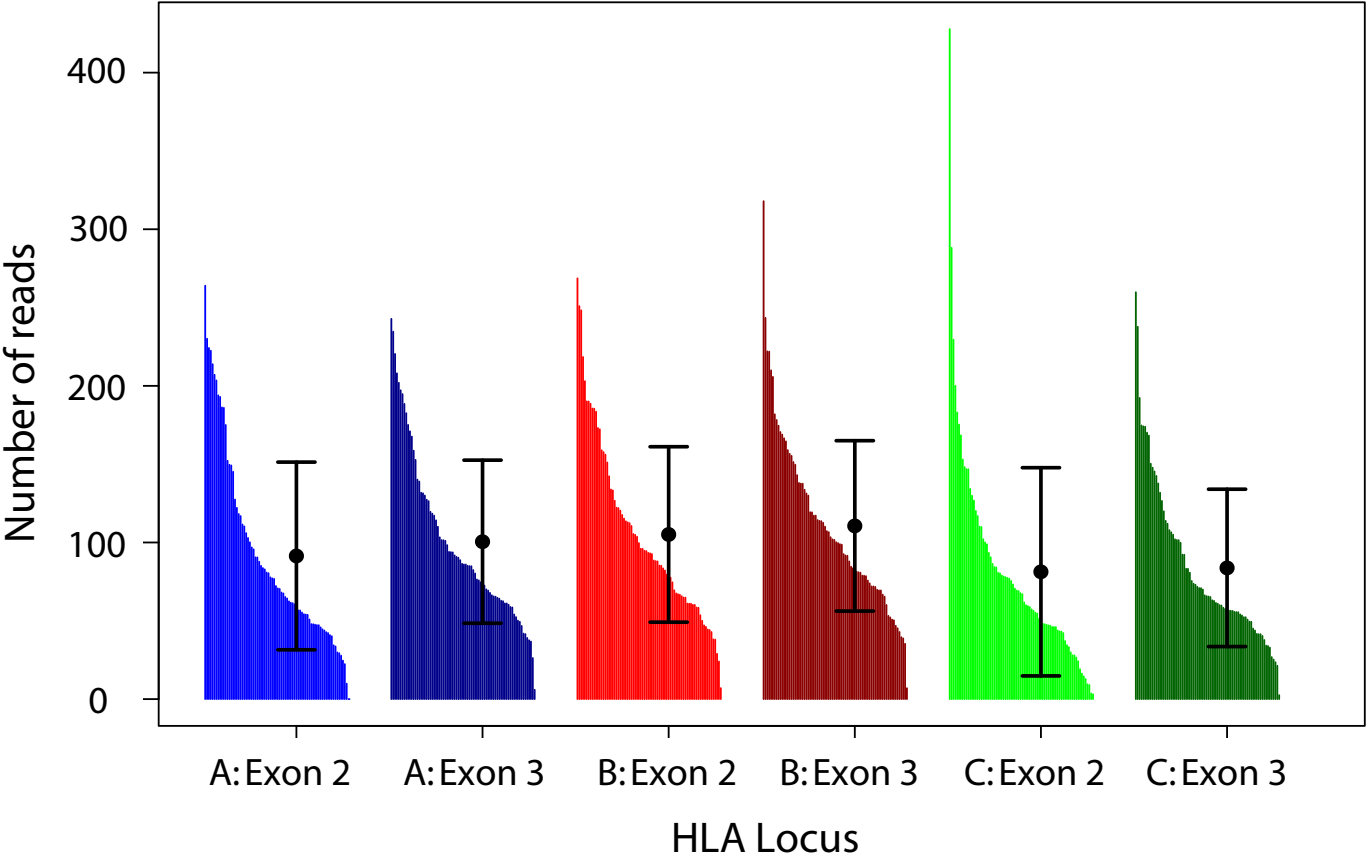

Supplement: Additional file 5 — Depth of Coverage Plate 3. Depth of coverage for 96 of 270 HapMap individuals sequenced on plate 3: (a) coverage by individual, and (b) coverage across each of 6 exons. [file 1471-2164-12-42-S5.PDF]
