# Supplementary material for: Next-generation sequencing for HLA typing of class I loci
Source: BMC Genomics. 2011 Jan 18;12:42. doi: 10.1186/1471-2164-12-42 (PMC3033818; doi:10.1186/1471-2164-12-42)
Supplement: Additional file 8 — Example of mistyping. IGV snapshot: an example of mistyping at HLA-A due to low 454 coverage of A*3001 (NA18507). [file 1471-2164-12-42-S8.PDF]

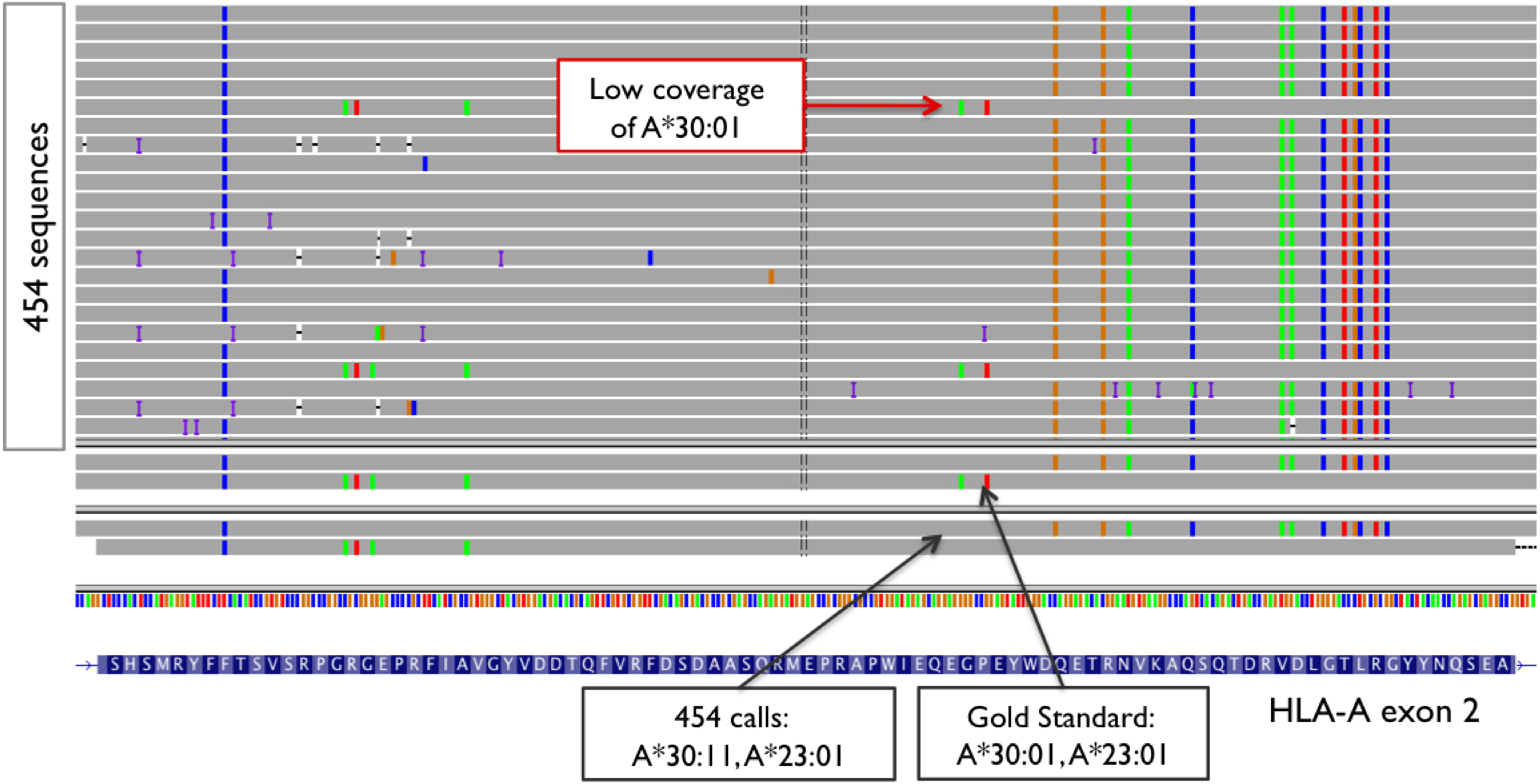
454 sequences
Low coverage of A*30:01
S H S M R Y F F T S V S R P G R G E P R F I A V G Y V D D T Q F V R F D S D A A S Q R M E P R A P W I E Q E G P E Y W D Q E T R N V K A Q S Q T D R V D L G T L R G Y Y N Q S E A
454 calls: A*30:11, A*23:01
Gold Standard: A*30:01, A*23:01
HLA-A exon 2
